# Choices of measures of association affect the visualisation and composition of the multimorbidity networks

**DOI:** 10.1186/s12874-024-02286-3

**Published:** 2024-07-23

**Authors:** Mohammad Reza Baneshi, Annette Dobson, Gita D. Mishra

**Affiliations:** Australian Women and Girls’ Health Research Centre, School of Public Health, Faculty of Medicine, The University of Queensland, Level 3 Public Health Building, 288 Herston Rd, Herston, QLD 4006 Australia

**Keywords:** Pattern finding, Network analysis, Strength of association, Community, Weight

## Abstract

**Background:**

Network analysis, commonly used to describe the patterns of multimorbidity, uses the strength of association between conditions as weight to classify conditions into communities and calculate centrality statistics. Our aim was to examine the robustness of the results to the choice of weight.

**Methods:**

Data used on 27 chronic conditions listed on Australian death certificates for women aged 85+. Five statistics were calculated to measure the association between 351 possible pairs: odds ratio (OR), lift, phi correlation, Salton cosine index (SCI), and normalised-joint frequency of pairs (NF). Network analysis was performed on the 10% of pairs with the highest weight according to each definition, the ‘top pairs’.

**Results:**

Out of 56 ‘top pairs’ identified, 13 ones were consistent across all statistics. In networks of OR and lift, three of the conditions which did not join communities were among the top five most prevalent conditions. Networks based on phi and NF had one or two conditions not part of any community. For the SCI statistics, all three conditions which did not join communities had prevalence below 3%. Low prevalence conditions were more likely to have high degree in networks of OR and lift but not SCI.

**Conclusion:**

Use of different statistics to estimate weights leads to different networks. For exploratory purposes, one may apply alternative weights to identify a large list of pairs for further assessment in independent studies. However, when the aim is to visualise the data in a robust and parsimonious network, only pairs which are selected by multiple statistics should be visualised.

## Background

Across the globe, higher life expectancy has led to an increase in the number of people living with multimorbidity. In epidemiological studies, multimorbidity is defined as the presence of two or more chronic medical conditions [1]. While better management of patients requires a deep understanding of the diseases that occur together more frequently [2], there is a need for methods to describe patterns of association among multimorbid conditions.

Network analysis is a popular pattern finding tool with practical appeal in the context of multimorbidity [3]. There is a distinction between a network as a visualisation tool and network analysis. A network is characterised as a set of nodes and edges, with weights showing the strength of association between two connected nodes [4]. Throughout this manuscript, the words weight and strength of association are used interchangeably. In the multimorbidity setting, conditions are treated as nodes and the pairwise association between several diseases are visualised as a network with an edge between two associated conditions.

On the other hand, network analysis is an analytical tool which uses the weight to classify the conditions into communities of densely connected conditions, with conditions belonging to different communities being weakly associated. Additionally, it provides measures of the centrality for conditions.

A current scoping review of papers published to describe patterns of multimorbidity using network analysis found heterogeneity in terms of methods used to calculate weight between conditions [5]. The aim of this paper was to apply alternative weights to examine whether use of different statistics resulted in the selection of different pairs, and differences in the composition of the networks.

## Methods

### Measure of association between conditions

Traditionally, the strength of association between conditions has been measured using the Pearson correlation coefficient for binary variables [6]. To reduce the complexity of the networks, only pairs of conditions which were correlated at significance level of 0.05 were visualised by a network. It has been argued that this measure cannot detect associations between rare conditions. Moreover, the number of ‘significant’ correlations, which influences the density of the network, is affected by the sample size [7]. Over the past few years, variety of other statistics such as odds ratio or relative risk have been considered as weight, and different thresholds or approaches applied to reduce the complexity [5, 8].

In this manuscript, five different statistics were calculated to describe the strength of association between each pair: Odds Ratio (OR), lift, phi correlation, Salton Cosine Index (SCI), and normalised joint frequency of pairs (NF).

Lift was calculated by dividing the proportion of subjects who had both conditions, by the product of proportion of subjects with each condition. The Salton Cosine Index (SCI) was calculated by dividing the observed joint frequency of each pair with the square root of product of frequency of corresponding conditions. To calculate the normalised joint-frequencies (NF), first the joint frequencies of all pairs were summarised in a symmetric matrix, known as the adjacency matrix. Entries on the main diagonal corresponded to the joint frequency of each condition with itself and therefore was set at zero. Then, to overcome the effect of the differing prevalence of the conditions, the Iterative Proportional Fitting (IFP) method is applied to the adjacency matrix so that all the row and column marginal frequencies were one [9].

### Dimension reduction

To reduce the complexity and to improve the interpretability of results, using each measure of association, only pairs with weight above the 90th percentile of the distribution of weights were regarded as being strongly associated and visualised in the networks (‘top pairs’).

#### Community detection

Using each of five weights, the conditions were classified into separate communities. Through an iterative procedure, network analysis classified the conditions into separate communities by maximising a statistic known as modularity [9]. The modularity of a network with a total of *m* edges is defined by Formula 1.

$$Formula1:Q = {1 \over {2m}}\mathop \sum \limits_{i,j} \left[ {{A_{ij}} - {\rm{\gamma }}{{{K_i}{K_j}} \over {2m}}} \right]\delta ({c_i},{c_j})$$

Where$\:{A}_{ij}\:$is the weight of the edge between nodes *i* and *j*,

$\:{K}_{i}$ and $\:{K}_{j}$ are the sums of weights of the edges attached to nodes *i* and *j*,

the expected number of edges between nodes *i* and *j* is

$${{{K_i}{K_j}} \over {2m}} = {{{K_i}{K_j}} \over {\mathop \sum \nolimits_{ij} {A_{ij}}}}$$

$\:{c}_{i}\:$and $\:{c}_{j}\:$are the communities to which nodes *i* and *j* are assigned,

$\:\delta\:=1$ if nodes are assigned to the same community, and 0 otherwise, and

$\:{\gamma\:}$ is the resolution parameter which controls the number of communities, where higher resolution leads to more communities.

The resolution parameter was set at 1 for all five networks. A method called the Leiden algorithm was applied to identify communities [10]. The algorithm assigned each node to a different community. The gain in the modularity statistic by removing node *i* from its community and putting it into community *j* was computed for all nodes. Node *i* was merged with the community for which the gain was maximal. The algorithm allowed for further split of communities into subcommunities. The process was applied to all nodes until no further improvement could be achieved.

### Centrality statistics

To demonstrate the effect of weight on centrality statistics, using each of five measures of association, for each condition, the centrality statistics of degree, closeness, and betweenness were calculated. For a specific condition X_1_, the degree is the sum of weights of all other conditions connected to X_1_. The closeness is the reciprocal of the sum of the length of the shortest paths between X_1_ and all other conditions. The betweenness for X_1_ is the sum of the proportion of shortest paths between other pairs of conditions that passes through condition X_1_.

### Sample and list of conditions

The data for all deaths in Australia from 2006 to 2018 were supplied by the Australia Coordinating Registry (*N* = 1,932,018). Death certificates are compiled using the underlying and contributing causes of death. The final list of conditions used for this paper included 27 chronic conditions with prevalence > 1%. The data used here were for women aged 85+. Only deaths with multimorbidity (i.e., underlying and one or more contributing conditions on the death certificate) which were certified by medical doctors were analysed (*N* = 283,195).

For the data analysis, the following R packages were used: ipfr to normalise the joint frequencies [11], and igraph to visualise the networks, detect communities, and calculate the centrality statistics [12].

## Results

Twenty-seven conditions with a prevalence range of 33.4% for ‘Dementia, Alzheimer’s disease’ to 1.5% for ‘eye, ear diseases’ were selected (Table 1), where lower rank indicates higher prevalence.

**Table 1:** Prevalence of the conditions listed anywhere on the death certificates for women in Australia aged 85 years or more who had more than one cause of death mentioned (*N* = 283,195)

Rank	Condition	Prevalence (%)
1	Dementia, Alzheimer’s disease	33.4
2	Ischemic Heart Disease	32.2
3	Hypertensive disease	26.7
4	Cerebrovascular disease	20.6
5	Other circulatory diseases	17.1
6	Heart failure	16.4
7	Cardiac arrhythmia	15.1
8	Kidney disease	14.5
9	Diabetes	12.5
10	Symptoms, signs, ill-defined conditions	11.1
11	Chronic lower respiratory disease	11.0
12	Musculoskeletal disease	10.4
13	Other digestive diseases	7.9
14	Infectious, parasitic diseases	6.7
15	Other malignant neoplasms	5.9
16	Other respiratory diseases	5.1
17	Psychiatric and other mental disorders	4.6
18	Influenza, pneumonia	4.4
19	Breast cancer	3.3
20	Colorectal cancer	3.1
21	Lymph, blood cancer	3.0
22	Other endocrine diseases	3.0
23	Other neurological conditions	2.2
24	Parkinson’s disease	2.2
25	Skin disease	2.0
26	Lung, tracheal cancer	1.5
27	Eye, ear disease	1.5

**Fig. 1:**
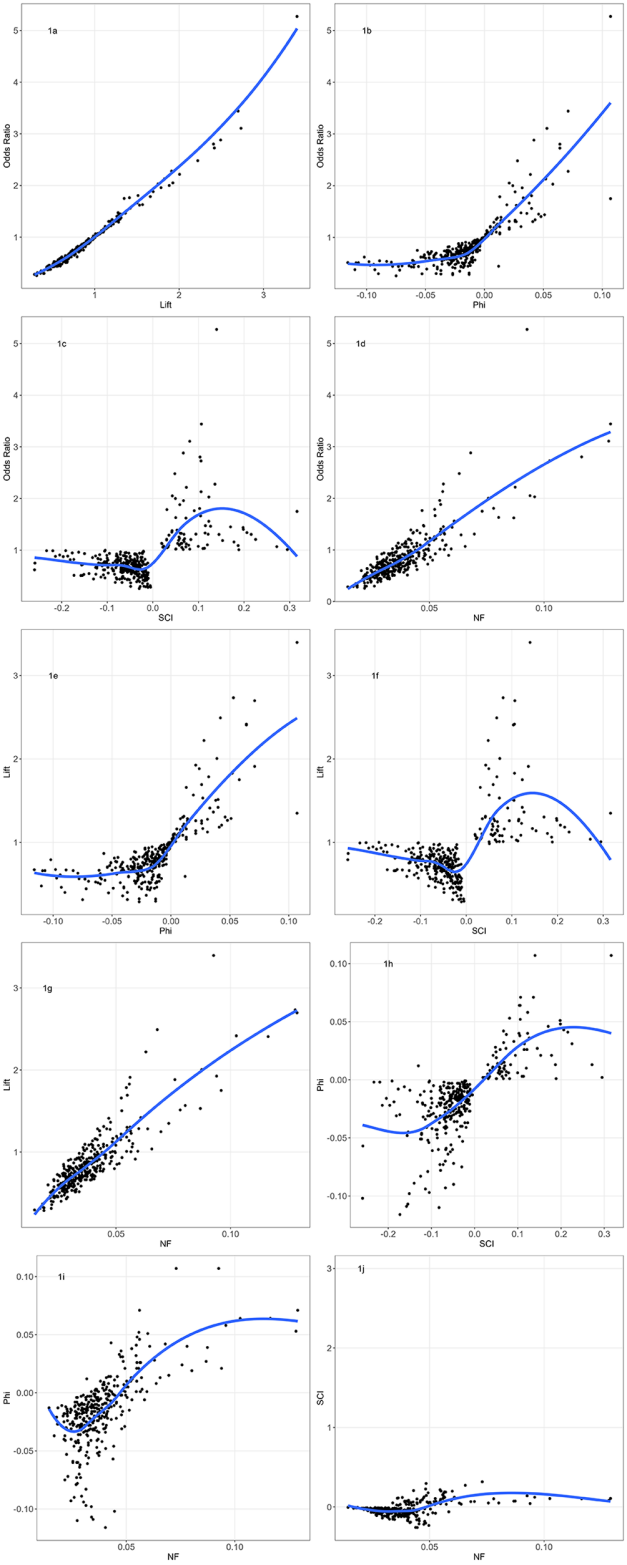
Scatterplot of different weights (for all 351 possible pairs) OR: Odds ratio, SCI: Salton cosine index, NF: normalised joint frequency; graphs are not on the same scale

Weights derived from OR, lift, and NF were approximately linearly associated (Fig. 1a, d, and g). The associations between the other statistics were more complicated (Fig. 1b, c, e, f, h, i, and j).

The total number of unique pairs selected as being strongly associated (‘top pairs’) by any measure was 56, with 13 pairs being selected by all five measures of association (Table 2). Ten pairs were selected only by one statistic (6 only by SCI and 4 only by NF).

**Table 2:** Pairs of conditions selected as being strongly associated by each of five measures of association

Pair	Conditions	OR	Lift	Phi	SCI	NF
1 + 4	Dementia, Alzheimer’s disease + Cerebrovascular disease	0	0	0	1	1
2 + 3	Ischemic Heart Disease + Hypertensive disease	0	0	0	1	0
3 + 4	Hypertensive disease + Cerebrovascular disease	1	1	1	1	1
3 + 7	Hypertensive disease + Cardiac arrhythmia	0	0	1	1	0
3 + 9	Hypertensive disease + Diabetes	0	0	1	1	0
4 + 5	Cerebrovascular disease + Other circulatory diseases	0	0	0	1	1
4 + 7	Cerebrovascular disease + Cardiac arrhythmia	0	0	0	1	1
5 + 7	Other circulatory diseases + Cardiac arrhythmia	0	0	1	1	0
6 + 7	Heart failure + Cardiac arrhythmia	1	1	1	1	1
6 + 8	Heart failure + kidney disease	1	1	1	1	1
8 + 9	Kidney disease + Diabetes	0	0	1	1	1
1 + 17	Dementia, Alzheimer’s disease + Psychiatric and other mental disorders	0	0	1	1	0
3 + 12	Hypertensive disease + Musculoskeletal disease	1	0	1	1	0
3 + 17	Hypertensive disease + Psychiatric and other mental disorders	0	0	0	1	0
6 + 11	Heart failure + Chronic lower respiratory disease	1	1	1	1	1
6 + 16	Heart failure + other respiratory diseases	1	0	1	1	1
8 + 14	Kidney disease + Infectious, parasitic diseases	0	0	1	1	1
8 + 18	Kidney disease + Influenza, pneumonia	0	0	0	0	1
3 + 22	Hypertensive disease + Other endocrine diseases	1	1	1	1	0
3 + 27	Hypertensive disease + Eye, ear disease	1	1	1	1	0
4 + 23	Cerebrovascular disease + Other neurological conditions	1	1	1	1	1
4 + 24	Cerebrovascular disease + Parkinson’s disease	0	0	0	0	1
10 + 12	Symptoms, signs, ill-defined conditions + Musculoskeletal disease	0	0	0	1	0
10 + 13	Symptoms, signs, ill-defined conditions + Other digestive disease	0	0	0	1	0
10 + 14	Symptoms, signs, ill-defined conditions + Infectious, parasitic diseases	1	1	1	1	1
10 + 16	Symptoms, signs, ill-defined conditions + Other respiratory disease	0	1	1	1	1
10 + 17	Symptoms, signs, ill-defined conditions + Psychiatric and other mental disorders	0	0	0	1	0
10 + 18	Symptoms, signs, ill-defined conditions + Infleunza, pneumonia	1	1	1	1	1
11 + 12	Chronic lower respiratory disease + Musculoskeletal disease	0	0	0	1	0
11 + 17	Chronic lower respiratory disease + Psychiatric and other mental disorders	1	1	1	1	0
12 + 13	Musculoskeletal disease + Other digestive disease	1	1	1	1	0
12 + 17	Musculoskeletal disease + Psychiatric and other mental disorders	1	1	1	1	1
14 + 18	Infectious, parasitic diseases + Infleunza, pneumonia	0	0	0	0	1
16 + 18	Other respiratory disease + Infleunza, pneumonia	1	1	1	0	1
10 + 27	Symptoms, signs, ill-defined conditions + Eye, ear disease	1	1	0	0	0
11 + 26	Chronic lower respiratory disease + Lung, tracheal cancer	1	1	1	1	1
12 + 22	Musculoskeletal disease + Other endocrine diseases	1	1	1	1	1
12 + 23	Musculoskeletal disease + Other neurological conditions	1	1	0	0	0
12 + 25	Musculoskeletal disease + Skin disease	1	1	0	0	0
12 + 27	Musculoskeletal disease + Eye, ear disease	1	1	1	1	1
13 + 27	Other digestive disease + Eye, ear disease	1	1	1	0	1
14 + 25	Infectious, parasitic diseases + Skin disease	1	1	1	0	1
15 + 19	Other malignant neoplasms + Breast cancer	1	1	1	1	1
15 + 20	Other malignant neoplasms + Colorectal cancer	1	1	1	1	1
15 + 26	Other malignant neoplasms + Lung, tracheal cancer	1	1	1	0	1
16 + 26	Other respiratory disease + Lung, tracheal cancer	0	1	0	0	1
17 + 22	Psychiatric and other mental disorders + Other endocrine diseases	1	1	1	0	0
17 + 23	Psychiatric and other mental disorders + Other neurological conditions	1	1	0	0	0
17 + 24	Psychiatric and other mental disorders + Parkinson’s disease	1	1	0	0	1
17 + 26	Psychiatric and other mental disorders + Lung, tracheal cancer	1	1	1	0	1
17 + 27	Psychiatric and other mental disorders + Eye, ear disease	1	1	1	0	1
19 + 20	Breast cancer + Colorectal cancer	1	1	0	0	1
19 + 21	Breast cancer + Lymph, blood cancer	0	0	0	0	1
22 + 27	Other endocrine diseases + Eye, ear disease	1	1	1	0	1
23 + 24	Other neurological conditions + Parkinson’s disease	1	1	1	0	1
23 + 27	Other neurological conditions + Eye, ear disease	1	1	0	0	0

1 the pair selected as top; 0: the pair did not select as top

The number of times three conditions with the lowest prevalence (i.e., rank 25 (skin disease), 26 (lung, tracheal cancer), and 27 (eye, ear disease) in Table 1) contributed to form ‘top pairs’ by each measure of association was 13 by OR, 13 by lift, 9 by phi, 3 by SCI, and 9 by NF. Corresponding numbers for three conditions with the highest prevalence (i.e., those ranked 1 (Dementia, Alzheimer’s disease), 2 (ischemic heart disease), and 3 (hypertensive disease) in Table 1) was 4 in OR, 3 in lift, 7 in phi, 10 in SCI, and 2 in NF.

Most of the pairs selected by OR and lift statistics were the same (33 out of 35), giving a Kappa for agreement of 0.85. The Kappa values for agreement between SCI and all other measures, except Phi, were negative suggesting they measure different constructs.

**Fig. 2:**
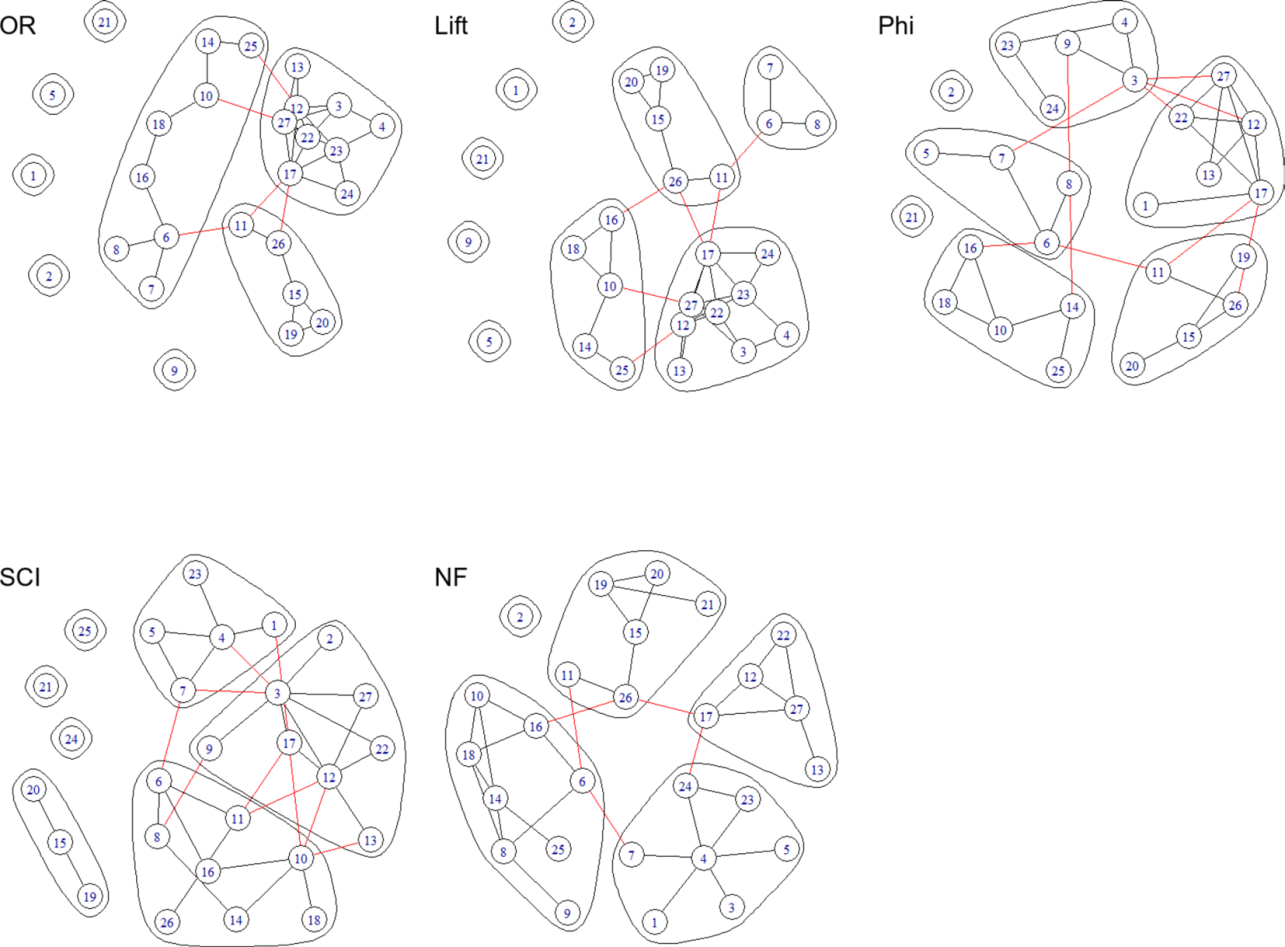
Communities identified in networks based on different weights: OR (top left), lift (top middle), Phi (top right), SCI (bottom left), and NF (bottom middle). Conditions 16 and 18 are respiratory diseases. Conditions 15, 19, 20, 21, and 26 are different types of cancers. Conditions 3, 4, 5, 6, and 7 are cardiovascular conditions. Conditions 17, 23, and 24 are neurological conditions

Figure 2 shows the networks and communities obtained using each of the five measures of association. The total number of communities found in each of the five networks were 3 for OR, 4 for lift, 5 for Phi, 5 for SCI, and 4 for NF. In the networks based on OR and lift, four conditions ranked below nine (i.e., ranked after diabetes with prevalence below 12.5%) in Table 2 did not join other communities. All three conditions that did not join other communities in the SCI network all had ranks above 21 in Table 2 (i.e., ranked before lymph, blood cancer with prevalence above 3%). On the other hand, in the network based on NF the condition ranked two on prevalence list in Table 2 (i.e., ischemic heart disease) was not in any community.

The grouping showed reasonable clinical coherence. For example, all five metrics put respiratory conditions of infleunza, pneumonia (rank 18) and other respiratory disease (rank 16) into one community. The SCI metric divided the cancer related conditions into three separate communities. The other four metrics classified the cancer into one community: breast cancer (rank 19 in Table 1), colorectal cancer (rank 20), lymph blood cancer (rank 21), lung tracheal cancer (rank 26), and other malignant neoplasms (rank 15). The OR, Lift, Phi, and NF metrics divided the cardiovascular conditions of hypertensive disorders (rank 3), cerebrovascular disease (rank 4), heart failure (rank 6), and cardiac arrhythmia (rank 7) into two community. OR, Lift, and Phi metrics categorised the neurological conditions of psychiatric and other mental disorders (rank 17), other neurological conditions (rank 23), and Parkinson’s disease (rank 24) into one single community. The NF metric put these conditions into two communities with a link between them (through conditions 17 and 24).

There were differences between the measures of association in terms of which conditions had the highest degree, closeness and betweenness. The top three conditions with the highest value for each measure are shown in Table 3. For example, condition 27 (i.e., eye, ear disease) had high degree in networks of OR, lift, and Phi but not in networks of SCI and NF, suggesting that the association between this condition and other conditions was more likely to be captured by OR, lift, and Phi measures of association.

**Table 3:** The three conditions (as ranked in table 1) with the highest values for degree, closeness and betweenness based on each of the five measures of association

Condition (Rank in Table 1)	Degree					Closeness					Betweenness				
	OR	Lift	Phi	SCI	NF	OR	Lift	Phi	SCI	NF	OR	Lift	Phi	SCI	NF
Hypertensive disease (3)			✓	✓				✓	✓				✓	✓	
Cerebrovascular disease (4)				✓	✓										✓
Heart failure (6)								✓					✓		
Symptoms, signs, ill-defined conditions (10)														✓	
Chronic lower respiratory disease (11)						✓					✓	✓			
Musculoskeletal disease (12)	✓	✓	✓	✓										✓	
Other malignant neoplasms (15)					✓										
Other respiratory disease (16)										✓					
Psychiatric and other mental disorders (17)	✓	✓					✓	✓		✓	✓	✓	✓		✓
Breast cancer (19)									✓						
Colorectal cancer (20)									✓						
Lung, tracheal cancer (26)					✓	✓	✓			✓	✓	✓			✓
Eye, ear disease (27)	✓	✓	✓			✓	✓								

## Discussion

The aim of this paper was to examine the influence of different weights on the structure of a network. A recent scoping review of methods for analysing patterns of multimorbidity using network analysis found that several techniques were used to measure the strength of association between conditions including the correlation coefficient, odds ratio, lift, and Salton Cosine Index (SCI) [5]. Additionally, the normalised joint frequency of pairs of conditions was used as a weight to visualise the multimorbidity network in the Italian population [9]. Therefore, we applied these five weights to examine their effects on the composition of the networks. For illustration, the weights between 351 pairs of chronic health conditions were calculated using five different statistics: OR, lift, Phi correlation, SCI, and normalised joint frequencies (NF).

Pairs with weight above the 90th percentile of the distribution were considered to be strongly associated and visualised. Under the hypothesis that the ‘different measures of association select the same pairs of conditions as being strongly associated’, the expected number of unique pairs would be about 35. However, there were 56 pairs meeting the criterion of being strongly associated on at least one measure, with only 13 pairs being strongly associated on all five measures. This example shows that the choice of measure of association will affect the identification of the associated pairs which are visualised by a network, and hence the central conditions and communities. Indeed, marked differences were seen between the five networks. For example, in the SCI network, some low prevalence conditions were not linked with other communities. In contrast, in the OR and lift networks, most conditions which did not merge into communities had high prevalence.

These findings illustrate that to produce robust results, an analysis of co-occurrences of nodes (in this case chronic conditions) should involve multiple measures of association.

In the literature some studies have not provided details of the weights used for their network analysis [13]. Other studies using network analysis have used only one weight. Furthermore, methods applied to reduce the complexity of the networks have differed. For example, a comorbidity network for type 2 diabetes mellitus was visualised for pairs of conditions with OR > 1.2 and P-values for OR < 1e-5 [14]. Another study used OR > 1.2 or OR < 0.8 (for positive and negative associations) with P-values < 1e-5 to depict a comorbidity network of hypothyroidism in adults [15]. A comorbidity network for people living in rural Uganda was visualised for pairs of conditions which satisfied the following three conditions: RR > 1, Phi correlation > 0, and false discovery rate < 0.05. The last condition was applied to control the inflation in type one error due to multiple comparisons. In another study, pairs of conditions with lift > 1 were visualised as the comorbidity network of diseases related to obesity [16]. Other studies have used lift > 2 to indicate a strong association [17–19].

It has been argued that the lower and upper limits of the lift depends on the prevalence of conditions [20]. To overcome this shortcoming, Hernandez et al. used the standardised lift, which varies between 0 and 1, to visualise the multimorbidity network in the Irish population where values above 0.2 were considered as strong, [2].

SCI was used as the weight to examine the health disparities by gender [7]. In that study, the results using SCI were compared with results using Pearson correlations to select strong pairs. Using the Pearson correlations, the authors found that 14,463 pairs were associated with a P-value < 0.01. On this basis a cut-off value of 0.04 was applied to SCI to get similar number of pairs. Egidi et al. used normalised joint frequency as weight and defined pairs with weight above the 95th percentile as strongly associated [9].

Some studies applied a different method which performed both steps (i.e., the estimation of weights and the selection of the strongest associations to reduce the complexity) together through multivariable regression modelling. To visualise the network of depression and anxiety symptoms, each condition in turn was treated as the outcome and all other conditions were treated as predictors [21]. Lasso logistic regression was applied to estimate regression coefficients (i.e., weights) and identify strong associations [21]. Therefore, weight derived from this method was adjusted after control of other conditions. As the Lasso method estimated adjusted measures of association and other methods compute a univariate measure, the Lasso method was not applied in this manuscript.

The purpose of the paper was to compare the effect of different measures of association on the composition of multimorbidity networks. To illustrate the methods, we used data from a subpopulation with a lot of multimorbidity. The composition of networks would be expected to be different for other age groups or for men instead of women.

This study had some limitations. First, we did not have an underlying model therefore it is difficult to judge which weight provided the most valid results. Second, there is no clear-cut way to distinguish between low, mid-range, and high prevalence conditions. Some of our inferences was based on the prevalence ranking of conditions.

## Conclusion

In conclusion, use of different statistics to estimate weights leads to different networks. We do not recommend any particular weight as the best for all data sets and research questions. For exploratory purposes, one may apply alternative weights to identify a large list of pairs for further assessment in independent studies. However, when the aim is to visualise the data in a robust and parsimonious network, only pairs which are selected by multiple statistics should be visualised.

## Data Availability

All R codes used in this article and joint frequencies of all pairs of conditions are available on the following GitHub page: https://github.com/rbaneshi2/patterns-among-binary-variables.

## Abbreviations

OR: Odds ratio
SCI: Salton cosine index
NF: Normalised joint frequency
